# CRISPR–Cas9 Based Bacteriophage Genome Editing

**DOI:** 10.1128/spectrum.00820-22

**Published:** 2022-07-26

**Authors:** Xueli Zhang, Chaohui Zhang, Caijiao Liang, Bizhou Li, Fanmei Meng, Yuncan Ai

**Affiliations:** a State Key Laboratory for Biocontrol, School of Life Sciences, Sun Yat-sen University, Guangzho, People’s Republic of China; University of California, San Diego

**Keywords:** bacteriophages, CRISPR-Cas9, genome editing, genome engineering

## Abstract

Bacteriophages are the most abundant entities in the biosphere, and many genomes of rare and novel bacteriophages have been sequenced to date. However, bacteriophage functional genomics has been limited by a lack of effective research methods. Clustered regularly interspaced short palindromic repeat/CRISPR-associated gene (CRISPR–Cas) systems provide bacteriophages with a new mechanism for attacking host bacteria as well as new tools for study bacteriophage functional genomics. It has been reported that bacteriophages are not only the driving elements of the evolution of prokaryote CRISPR arrays but also the targets of CRISPR–Cas systems. In this study, a phage genome editing platform based on the heterologous CRISPR–Cas9 system was theoretically designed, and a Vibrio natriegens phage TT4P2 genome editing experiment was carried out *in vivo* in the host bacterium Vibrio natriegens TT4 to achieve phage gene deletion and replacement. The construction of this phage genome editing platform is expected to solve the problem of insufficient research on phage gene diversity, promote the development of phage synthetic biology and nanotechnology, and even accelerate the discovery of new molecular biology tools.

**IMPORTANCE** Bacteriophages are the most numerous organisms on earth and are known for their diverse lifestyles. Since the discovery of bacteriophages, our knowledge of the wider biological world has undergone immense and unforeseen changes. A variety of *V. natriegens* phages have been detected, but few have been well characterized. CRISPR was first documented in Escherichia coli in 1987. It has been reported that the CRISPR–Cas system can target and cleave invaders, including bacteriophages, in a sequence-specific manner. Here, we show that the construction of a phage genome editing platform based on the heterologous CRISPR–Cas9 system can achieve *V. natriegens* phage TT4P2 gene editing and can also improve the efficiency and accuracy of phage TT4P2 gene editing.

## INTRODUCTION

Bacteriophages (phages) are unique organisms on Earth (1–6). The functions of most open reading frames (ORFs) in phage genome sequences in the GenBank database are currently unknown (7). Limited by gene editing technology and other factors, the genomes of approximately 66 *Vibrio* phages have been sequenced, but most research on functional genomics has stagnated at homology comparisons, causing studies on the functional genomics of *Vibrio* phages to lag behind the discovery of new phages and the sequencing of their genomes (8–10). At present, phage genome editing strategies can be classified into four categories: *in vitro* oligonucleotide (oligo) splicing, homologous recombination-mediated *in vivo* PCR DNA fragment splicing, endogenous and exogenous homologous recombination system-mediated editing, and jointly mediated editing by clustered regularly interspaced short palindromic repeat/CRISPR-associated gene (CRISPR–Cas) systems and homologous recombination.

First, because chemical synthesis capabilities are insufficient for the length of phage genomes, gene editing methods based on the chemical or biochemical synthesis of ssDNA or dsDNA (such as PCR)→DNA fragment splicing have been developed. Smith (11), Ando (12) and Gibson (13) used similar methods to complete the *in vitro* assembly of bacteriophage ϕX174 and the *in vivo* assembly or editing of bacteriophages such as T7. Second, most phage genome editing methods (such as point mutations, frameshift mutations, fragment insertions, gene deletions, and gene replacements) are based on small-scale gene mutations, but due to factors such as single phenotypes and a lack of selectable markers, it is difficult to screen target phage recombinants. Therefore, based on the original recombination system of the host bacteria (such as RecA/RecBCD), the commonly used foreign phage recombination system has been introduced to improve the efficiency of recombination. Marinelli (14) applied the bacteriophage recombineering of electroporated DNA (BRED) strategy to edit a series of mycobacteriophage genes and selected mycobacteriophage Che9c RecE/T protein gp60-61 to improve the efficiency of recombination. However, Oppenheim (15), Feher (16) and Shin (17) chose the Red system for use with the BRED strategy to edit phage genes. However, the gene editing efficiency of the BRED strategy is not high. The single plaque obtained by the method is mostly a mixture of wild-type and recombinant phages and accounts for only 1% to 22% of the total plaques.

Clustered regularly interspaced short palindromic repeat/CRISPR-associated gene (CRISPR–Cas) systems, which are adaptive immunity systems, are mainly encoded by prokaryotes (45% of sequenced eubacteria and 87% of sequenced archaea) (18). In addition, CRISPR–Cas systems with activity or potential activity have also been found in phages (Vibrio cholerae ICP1-related phage (19) and Cyanophage N-1) (20). The immune process of the CRISPR–Cas system can be divided into three steps: adaptation, expression and interference (21–27). Studies on CRISPR–Cas systems have promoted the development of new genome editing tools (26, 28–32). Genome editing technology based on the CRISPR–Cas system mainly uses two characteristics of interference processes: (i) base complementary pairing between crRNAs and the original spacer to locate the target sequence and (ii) the nuclease activity of specific Cas proteins to cleave and degrade foreign nucleic acids (27, 33, 34).

Bacteriophages are not only the driving elements of the evolution of prokaryote CRISPR arrays but also the targets of CRISPR–Cas systems (25, 34–36); thus, various CRISPR–Cas systems (especially type I, which account for 60% of all CRISPR–Cas systems (37–38)) are expected to be used to edit phage genomes. Phage genome editing mediated by CRISPR–Cas and homologous recombination systems provides a novel approach for bacteriophage functional genomics research. This new method mainly uses endogenous or exogenous CRISPR–Cas systems to degrade nontarget recombinants and improve the proportion of target recombinants. Phage genome editing based on endogenous CRISPR–Cas systems has been presented by Martel and Moineau (39) and Bari (40), while phage genome editing based on exogenous CRISPR–Cas systems has been reported by Kiro (41), Box (42), Lemay (43), Tao (44), and Shen (45). It is worth mentioning that compared with the efficiency of phage gene editing mediated by endogenous and exogenous homologous recombination systems (1% ~ 22%), the efficiency of phage genome editing mediated by the combination of CRISPR–Cas and homologous recombination systems is very high. Almost all single plaques picked from the double-layered plate are target recombinants. Phage genome editing based on the CRISPR–Cas system provides a new direction for the study of phage genome editing and can promote research on phage functional genomics. The development of new methods to improve the efficiency and accuracy of gene editing is the frontier of advancing phage genomics research.

**FIG 1:**
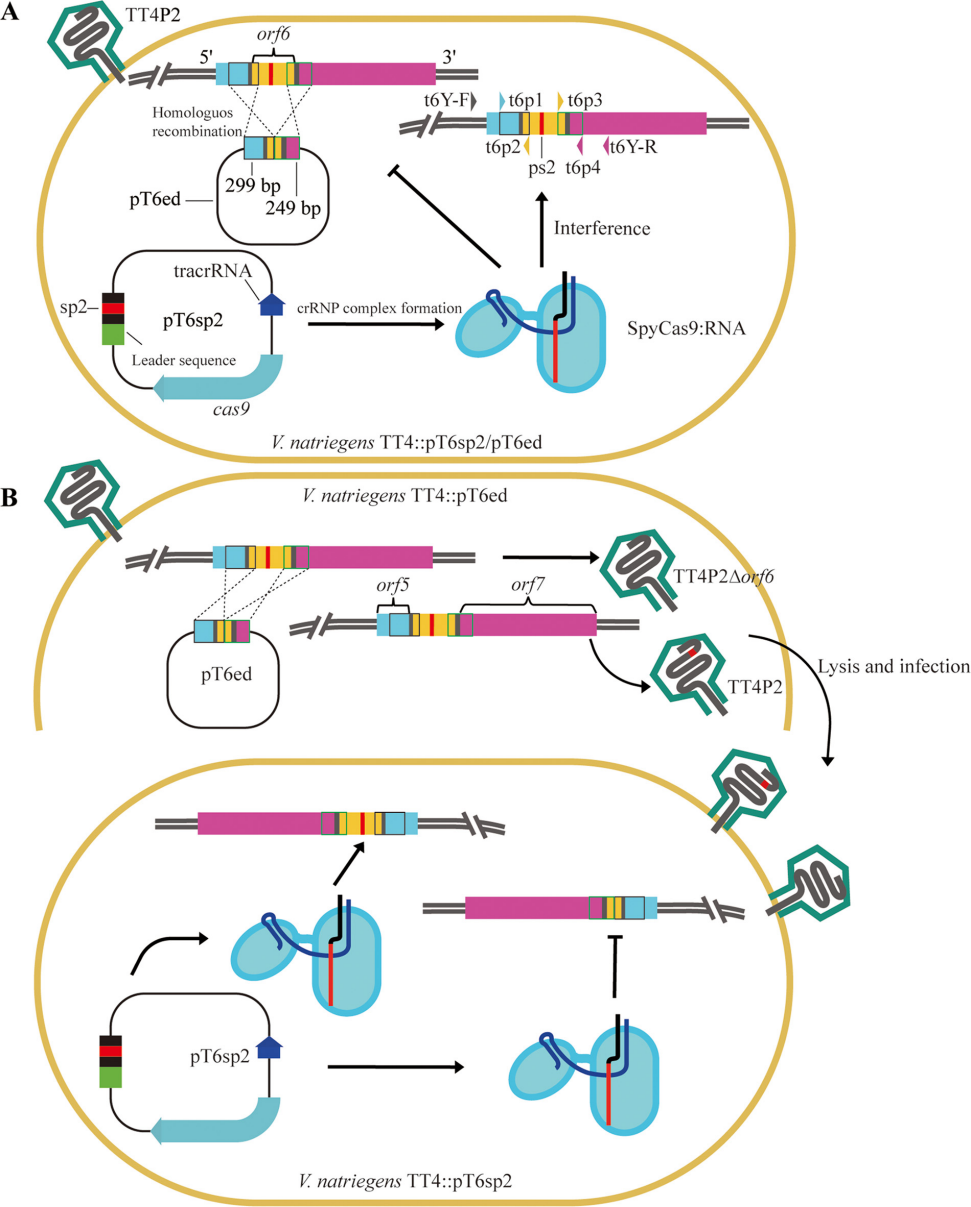
Two strategies for Vibrio natriegens phage genome editing based on the heterologous CRISPR–Cas9 system. The editing of phage TT4P2-*orf6* serves as an example to illustrate the two strategies. *V. natriegens* phage TT4P2-*orf6* is indicated by T6; TT4P2-*orf6*-spacer is indicated by T6sp, and it was connected to plasmid pCas9 to form plasmid pT6sp; TT4P2-*orf6*-editing is indicated by T6ed, and it was connected to plasmid pCRISPR to form plasmid pT6ed, and the primers used were t6Y-F/R, t6p1/p2 and t6p3/p4. ps is protospacer. TT4P2 is the wild-type phage TT4P2. (A) Dual-plasmid-mediated synchronous recombination (DPMSR): Wild phage TT4P2 infected *V. natriegens* TT4 containing the targeted plasmid pT6sp2 and the homologous recombination plasmid pT6ed, namely, TT4::pT6sp2/pT6ed, which was used to perform a double-layered plate experiment and then to obtain recombinants. In this strategy, homologous recombination between the homologous recombination plasmid and phage TT4P2 and the degradation of phage TT4P2 containing ps2 and protospacer-adjacent motif (PAM) using the crRNP complex (SpyCas9:RNA) were carried out simultaneously. (B) Single-plasmid-mediated asynchronous recombination (SPMAR): First, *V. natriegens* TT4::pT6ed was infected with wild-type TT4P2 to carry out a double-layered plate experiment. Then, the obtained mixed single plaque was used to infect *V. natriegens* TT4::pT6sp2 to perform another double-layered plate experiment and to obtain recombinants. Homologous recombination and the degradation of TT4P2 containing ps2 and PAM were carried out separately.

*V. natriegens* TT4 and *V. natriegens* siphophage TT4P2 were recently isolated and purified from the Pearl River Estuary of the South China Sea. The previous work by our laboratory summarized and reviewed the homologous recombination *in vivo* genome engineering method and its application and limitations in phage genome editing (46). In this study, phage genome editing platform based on heterologous CRISPR–Cas9 systems were theoretically designed and termed “dual-plasmid-mediated synchronous recombination (DPMSR)” (Fig. 1A) and “single-plasmid-mediated asynchronous recombination (SPMAR)” (Fig. 1B). Then, the genome of *V. natriegens* phage TT4P2 was edited *in vivo* in the host bacterium *V. natriegens* TT4 to verify the feasibility of the two theoretical design strategies. Finally, 292 bp and 162 bp of the *orf6* and *orf45* genes of the *V. natriegens* phage TT4P2, respectively, were deleted. The 292 bp DNA fragment of *orf6* was also successfully replaced with *orf39* of the Enterobacter myophage EJ(3)9P3, which encodes lysozyme *e*. The construction of a phage genome editing platform based on the heterologous CRISPR–Cas9 system provides a reference for the genome editing of *Vibrio* phages and lays the foundation for the next steps in the deep transformation of the phage genome (simplification of the phage genome and identification of dominant phage genes to construct multifunctional phages) (47).

## RESULTS

### Construction of the heterologous CRISPR–Cas9 gene editing system in *V. natriegens* TT4.

**(i) Identification of phage-resistant strains.** Genomic analysis revealed that the phage TT4P2 genome harbored no anti-CRISPR genes or innate CRISPR–Cas systems. Therefore, we chose to introduce the heterologous CRISPR–Cas9 system into TT4, which lacks a native CRISPR–Cas system, and attempted to construct an active system for high-efficiency genome editing of the phage TT4P2.

The editing efficiency of the heterologous CRISPR–Cas9 system depended on the construction of the recombination template strain and the phage-resistant strain. Therefore, the targeted plasmid pTorf-sp was electroporated into TT4 to construct phage-resistant strains that evade phage TT4P2 infection. Using the primers DR-F/R (Table S5 in the supplemental material), phage-resistant strains were amplified by PCR and sequencing, and the results indicated that the spacers of the targeted gene were linked to the plasmid pCas9. We successfully acquired the phage-resistant strain TT4::pTorf-sp (Table S1).

**(ii) Identification of recombination template strains (editing strains).** The recombination template plasmid pTorf-ed was introduced into TT4 via electroporation to construct recombination template strains to increase the recombination rate. Using the primers torf-p1/p4 (Table S5), the strains were verified by PCR (Fig. S1A–C) and sequencing, and the results indicated that the strains were successfully transformed with the plasmid pTorf-ed. We successfully acquired the recombination template strain TT4::pTorf-ed (Table S1).

**(iii) Identification of double-plasmid strains.** After obtaining *V. natriegens* TT4 containing a single plasmid, we also extracted the plasmids in TT4::pT6ed and TT4::pT45ed separately and then electrotransformed them into TT4::pT6sp2 and TT4::pT45sp1 competent cells. The strains were verified by PCR (Fig. S1D in the supplemental material) and sequencing, and the results indicated that we successfully acquired the double-plasmid strains TT4::pT6sp2/pT6ed and TT4::pT45sp1/pT45ed (Table S1).

**FIG 2:**
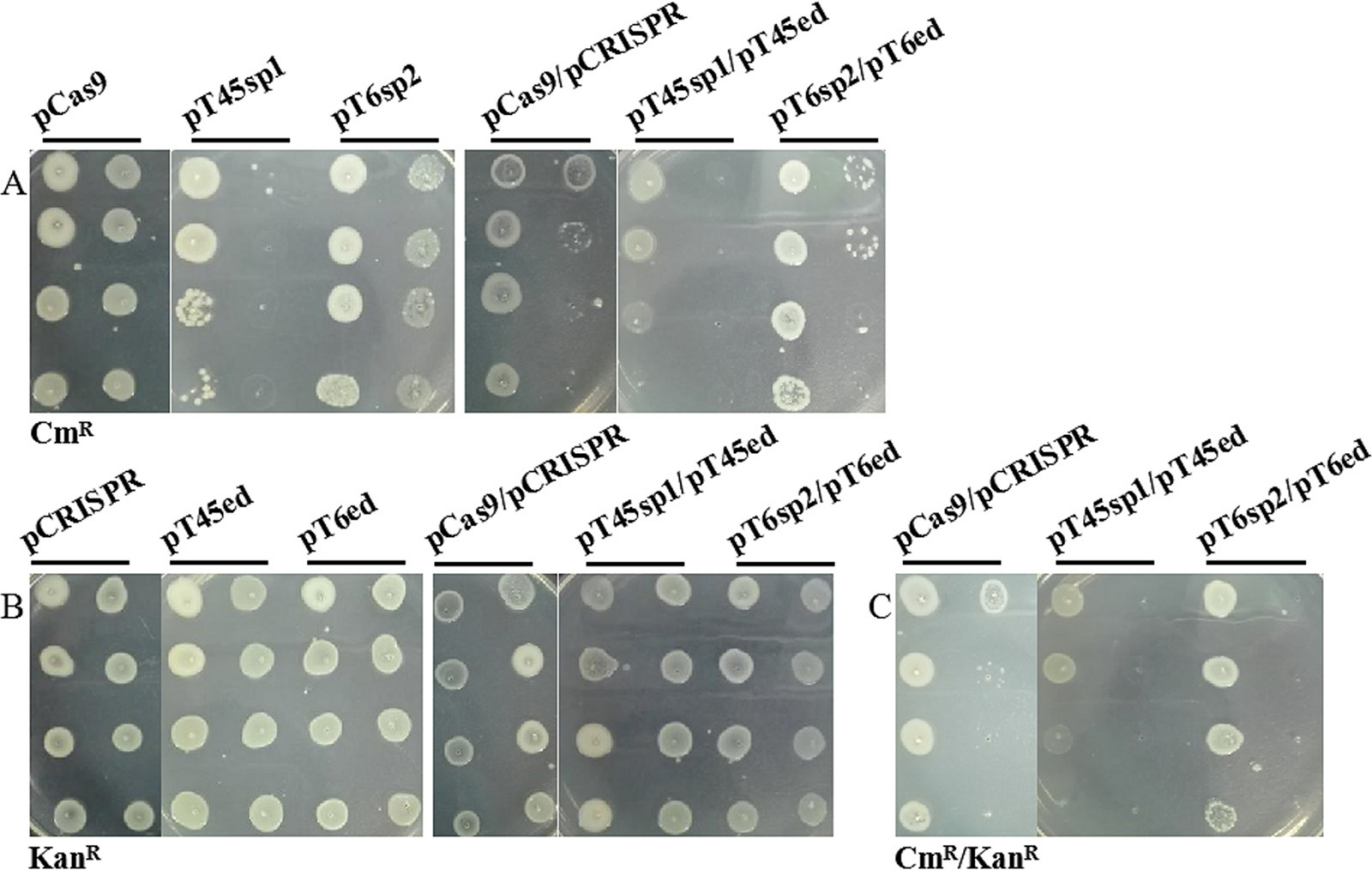
Stability of foreign plasmids in *V. natriegens* TT4. The foreign plasmids were electroporated into *V. natriegens* TT4. The displayed order (from top to bottom and from left to right) of the bacterial suspension of each strain is the 0^th^ to 7th generations. pT45sp1, pT45ed, pT6sp2, and pT6ed are the names of plasmids as annotated in Fig. 1. sp1 refers to the first spacer sequence of the targeted gene, and sp2 refers to the second spacer sequence of the targeted gene. (A) Stability of pCas9-type plasmids revealed by chloramphenicol resistance. Cm^R^ represents the chloramphenicol resistance of the strains containing pCas9-type plasmids. (B) Stability of pCRISPR-type plasmids revealed by kanamycin resistance. Kan^R^ represents the kanamycin resistance of the strains containing pCRISPR-type plasmids. (C) Stability of two plasmids revealed by Cm^R^/Kan^R^. Cm^R^/Kan^R^ represents the chloramphenicol and kanamycin resistance of the strains containing two plasmids.

**(iv) Stability of foreign plasmids in *V. natriegens* TT4.** To ensure the editing efficiency of the heterologous CRISPR–Cas9 system, referring to the method of Garneau and Hynes, the stability of strains containing the foreign plasmid was analyzed before the gene editing experiment. When TT4 contained only pCas9 or its derivative plasmids, the resistance to chloramphenicol lasted until the 7th generation or 4th generation (Fig. 2A). When TT4 simultaneously contained two plasmids, the duration of resistance to chloramphenicol was similar (Fig. 2C). However, whether the strains contained pCRISPR or its derivative plasmids or two plasmids, they always maintained strong kanamycin resistance at the 7th generation (Fig. 2B). The results showed that the stability of pCas9-type plasmids was weaker than that of pCRISPR-type plasmids in TT4. The reason may be that the copy number of pCRISPR is higher than that of pCas9. Therefore, the stability of the two-plasmid strain depends on the pCas9-type plasmid.

In addition, the strains containing stable plasmids (7th or 4th generations) were cultivated again, and then PCR (Fig. S5A in the supplemental material) was carried out with the corresponding primers using the suspension of each bacterium as the substrate. The resistance plasmids in various strains were mutated because the target band (814 bp) of the pCas9-type plasmids could be amplified with the primers pCas9-F/R1, but the target band (273 bp) could be amplified with the primers DR-F/R only in pT45sp1, while pCRISPR-type plasmids did not produce any band with the primers pCRISPR-F/R. Based on the above results, when phage TT4P2 genome editing was carried out, fresh 1st or 2nd generation suspensions of TT4 containing targeted plasmids or homologous recombination plasmids were used in this study.

**FIG 3:**
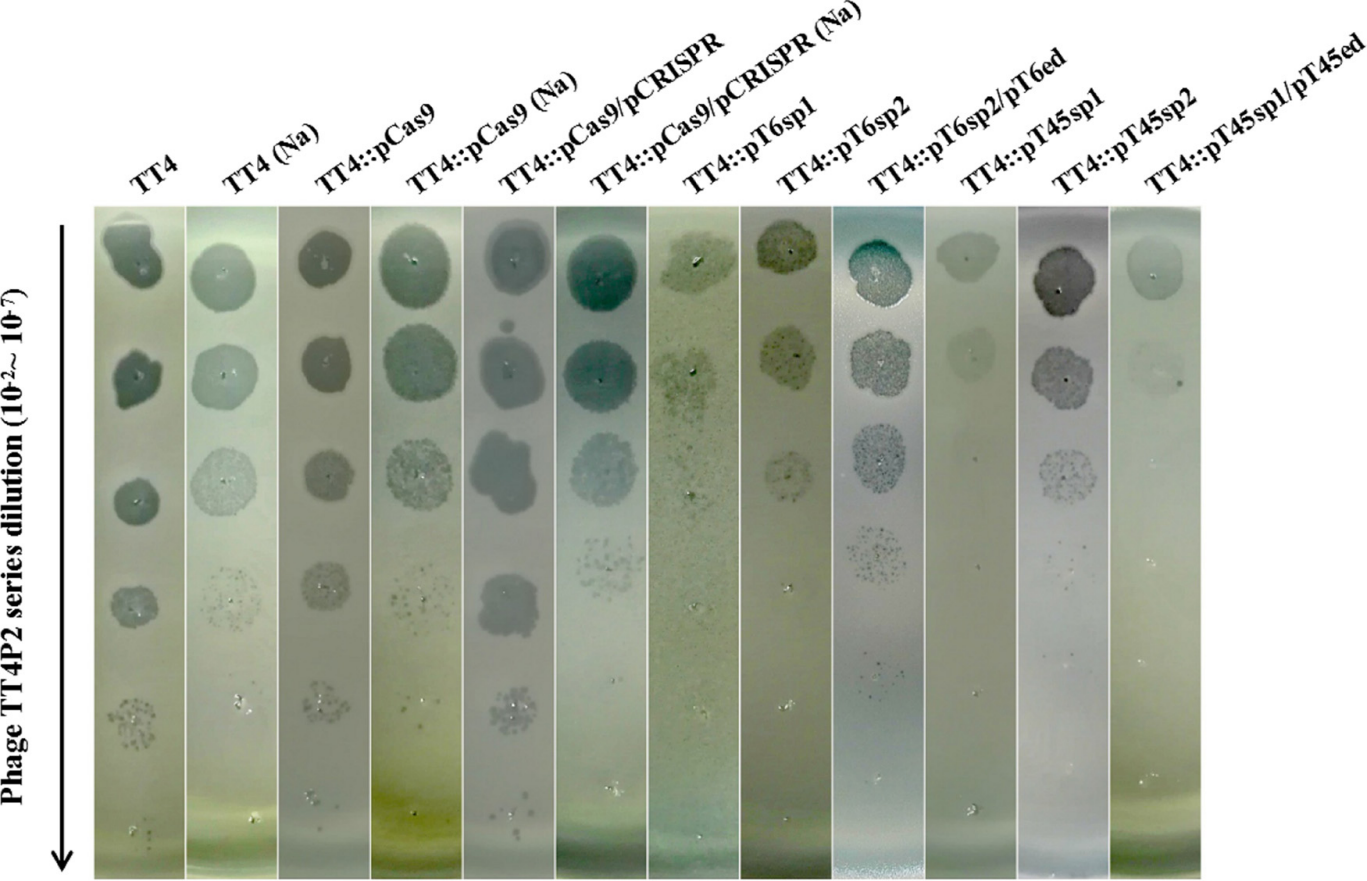
Immunity of *V. natriegens* TT4 containing the heterologous CRISPR–Cas9 system to phage TT4P2. A series dilution (10^−2^ ~ 10^−7^) of phage TT4P2 was spotted onto double-layered plates containing different indicator bacteria (marked at the top) to observe the resulting number of plaques. Then, according to the variation in plaque formation, whether phage TT4P2 was degraded by the CRISPR–Cas9 system was determined. Locations marked with Na indicate that the medium used for the double-layered plate was LB + 1% NaCl, and locations without Na indicate that the medium used was LB + 1% NaCl + 62.74 mM MgCl_2_.

**(v) Immunity of *V. natriegens* TT4 containing the heterologous CRISPR–Cas9 system to phage TT4P2.** After successfully constructing the heterologous CRISPR–Cas9 system in *V. natriegens* TT4, serial dilutions of phage TT4P2 were spotted onto a double-layered plate to determine the immunity of the system (Fig. 3). Regardless of whether TT4 contained only pCas9 or both pCas9 and pCRISPR, the infectivity of phage TT4P2 was similar to that of phage TT4P2 toward TT4. This result indicated that when the sequence of the plasmid pCas9 is different from the genome of phage TT4P2, the introduction of the plasmid pCas9 does not affect the infectivity of phage TT4P2 toward its host bacteria. However, when the spacer of pCas9, which can be digested with the *Bsa*I restriction enzyme, was replaced with sp1/sp2 targeting the *orf* of phage TT4P2, the infectivity of phage TT4P2 for TT4 containing the plasmid pTorf-sp was decreased. Moreover, the addition of Mg^2+^ to the medium could not only promote the formation of plaques but also make the plaques increasingly brighter, making it easier to observe and pick the plaques.

**FIG 4:**
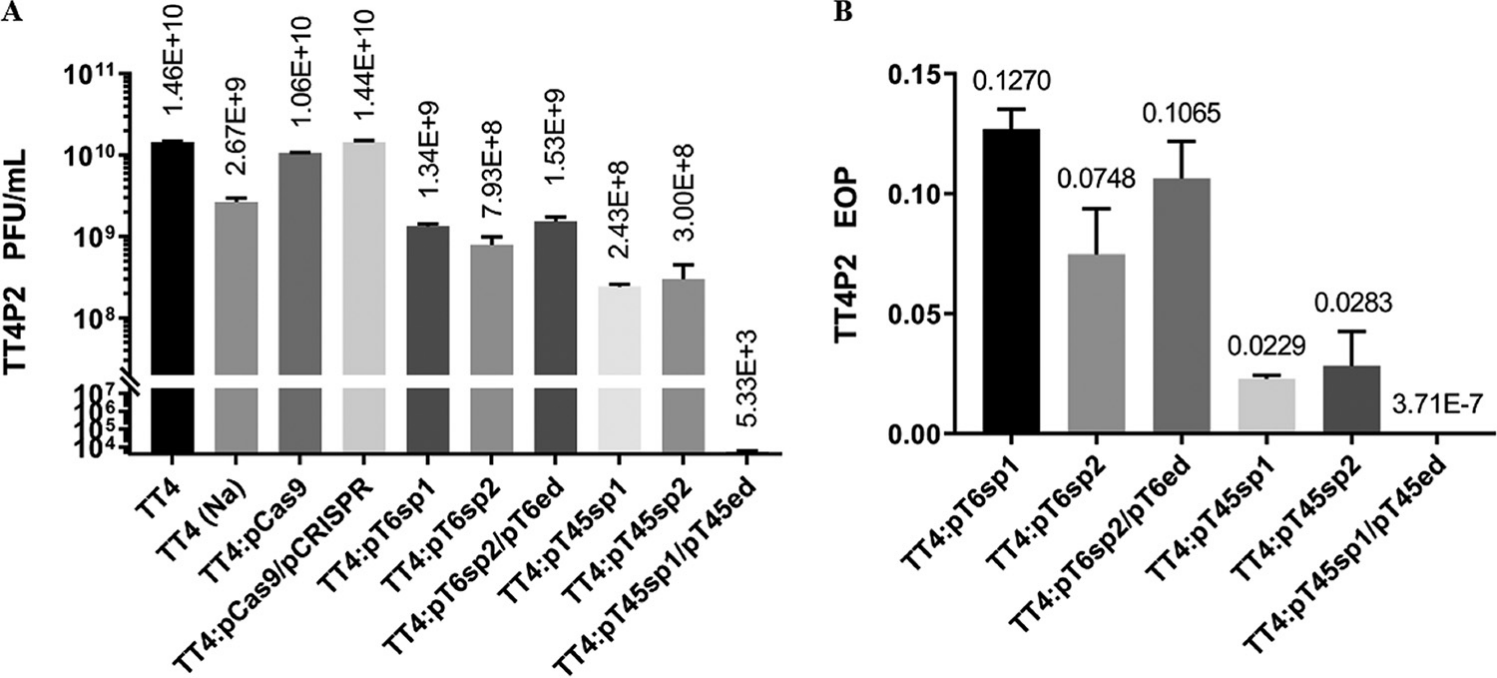
PFU and EOP of phage TT4P2. Locations marked with Na indicate that the medium used for the double-layered plate was LB + 1% NaCl, and locations without Na indicate that the medium used was LB + 1% NaCl + 62.74 mM MgCl_2_. The calculated PFU or EOP data were collected in three independent repeated double-layered plate experiments using the same materials. The control of TT4::pT6sp1, TT4::pT6sp2, TT4::pT45sp1 and TT4::pT45sp2 was TT4::pCas9. The control of TT4::pT6sp2/pT6ed and TT4::pT45sp1/pT45ed was TT4::pCas9/pCRISPR. The error bars represent the standard errors. (A) PFU of phage TT4P2. The wild-type phage TT4P2 was used to infect different strains containing foreign plasmids, and then the plaques on double-layered plates were counted. When counting the plaques, the final number of plaques was counted in all double-layered plates, except for TT4::pT45sp1/pT45ed, for which the large and middle plaques were included in the counts. (B) EOP of phage TT4P2. EOP = experimental group PFU/control group PFU.

Since the activity of SpyCas9:RNA in the strain was not static, the fresh strains had higher Cas9 activity in the experiment. For this reason, when phage TT4P2 infected strain TT4 containing the targeted plasmid, this experiment was also used to calculate the efficiency of plaquing (EOP, Fig. 4B) or plaque-forming units (PFU, Fig. 4A) of phage TT4P2. It can be seen from the figure that when TT4 contained targeted plasmids, it was extremely insensitive (*P < *0.01) to phage TT4P2 compared with the control. Therefore, if the selected targeted plasmids pTorf-sp were mostly intact and the SpyCas9:RNA activity was high, the immune effect on phage TT4P2 was better. In addition, when TT4::pT6sp2 was introduced into plasmid pT6ed, the PFU or EOP was increased; in contrast, after TT4::pT45sp1 was introduced into plasmid pT45ed, the PFU was decreased by 5 orders of magnitude. This result demonstrated that *orf45* may be more important than *orf6* in the life cycle of phage TT4P2.

### Gene editing of *V. natriegens* phage TT4P2.

**(i) Random mutant screening.** The heterologous CRISPR–Cas9 system constructed in *V. natriegens* TT4 is resistant to phages; thus, phages that have not been degraded by Cas9 may be either escape phages or mutant phages. To determine the types of these undegraded phages, *V. natriegens* TT4::pT6sp2 and *V. natriegens* TT4::pT45sp1 combined with appropriately diluted solutions of phage TT4P2 were used to carry out double-layered plate experiments. Six of 80 sequenced single plaques were picked in TT4::pT6sp2, and only one contained a point mutation at the PAM of the second protospacer (sp2) of *orf6*, which changed a Trp codon (TGG) to an opal stop codon (TGA), indicating that *orf6* may be a nonessential gene of phage TT4P2. The remaining 74 sequenced plaques were selected from TT4::pT45sp1, but none were mutants. These results indicated that perhaps the activity of SpyCas9:RNA in strain TT4 containing the plasmid pT45sp1 was not strong enough; thus, the escape phages that were not degraded by Cas9 in the double-layered plate were basically wild-type phages.

**FIG 5:**
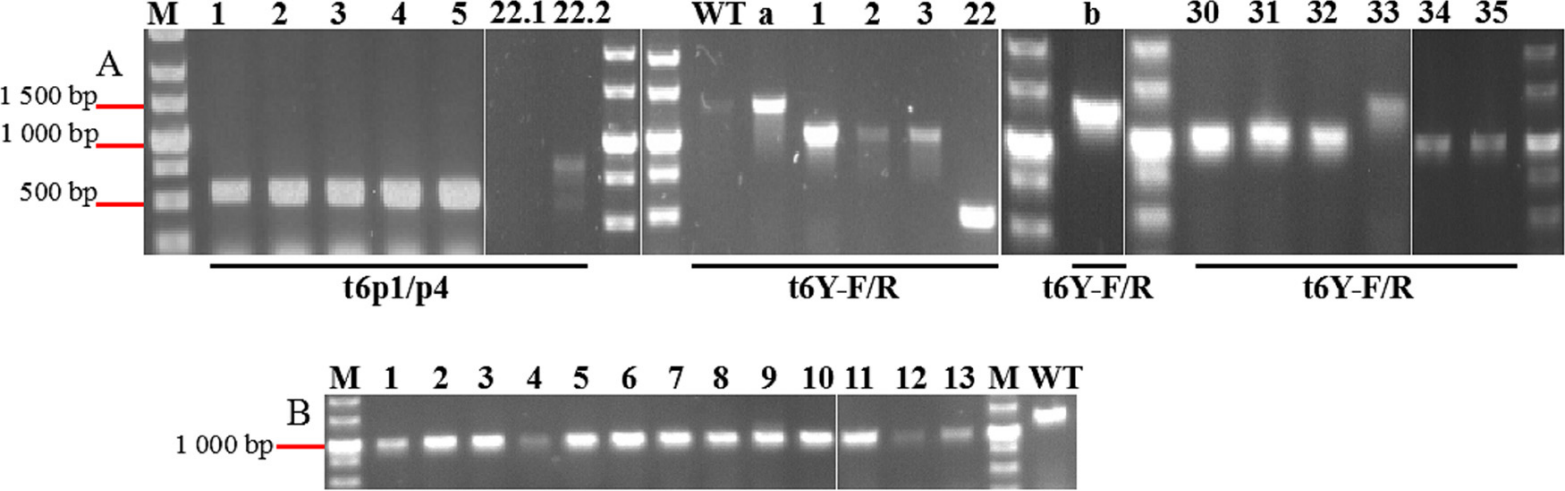
Deletion of phage TT4P2-*orf6* by two strategies based on the heterologous CRISPR–Cas9 system. M is the DL5000 DNA Marker, and WT is the wild-type phage TT4P2.The band was weak because the concentration of Na^+^ in the phage dilution was high, and the amount of phage dilution in the PCR system was 4.5 μL. The primers used were t6p1/p4 and t6Y-F/R. (A) PCR of partial plaques picked from double-layered plates obtained by the SPMAR method. Plaques 1–5, 22 and a were picked from double-layered plates with LB + 1% NaCl as the medium, and a is the parent of the first 29 plaques. Plaques 30–35 and b were picked from double-layered plates with LB + 1% NaCl + 62.74 mM MgCl_2_ as the medium, and b is the parent of plaques 30–35. All of these plaques were recombinants (deleted 292 bp) except for plaque 33, and plaque 22 was the nontarget recombinant, with 820 bp deleted. (B) PCR of partial plaques picked from double-layered plates obtained by the DPMSR method. All of the plaques were recombinants.

**(ii) Gene deletion.** After the heterologous CRISPR–Cas9 system was constructed in *V. natriegens* TT4, two strategies (DPMSR and SPMAR) were adopted for this platform to delete the partial sequence of *orf6*. Fifty-four plaques were picked from the double-layered plate, among which 35 plaques were obtained from the SPMAR method. According to the PCR (Fig. 5) and sequencing results, 34/35 plaques were recombinants, meaning that the editing efficiency of TT4P2-*orf6* by SPMAR was approximately 97%. The remaining 19 plaques obtained from DPMSR were all recombinants, meaning that the editing efficiency of TT4P2-*orf6* by DPMSR was approximately 100%. Moreover, the deletion of some undesired recombinants included the entire *orf6*, the back of *orf5* (121 bp), and the front of *orf7* (182 bp), which indirectly reflected that *orf5*, *orf6* and *orf7* are nonessential genes in the life cycle of phage TT4P2. The results show that both strategies based on the heterologous CRISPR–Cas9 system can successfully achieve the deletion of phage TT4P2-*orf6* and obtain the target homozygous recombinant TT4P2*Δorf6*, in which 292 bp was deleted.

**FIG 6:**
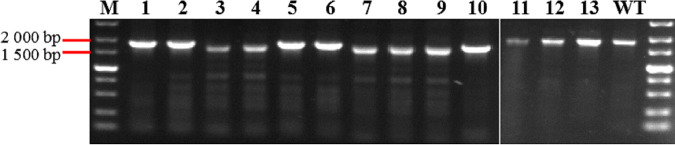
Deletion of phage TT4P2-*orf45* by the DPMSR method based on the heterologous CRISPR–Cas9 system. M is the DL5000 DNA Marker, and WT is the wild-type phage TT4P2. According to the PCR bands, plaques 3, 4, 7, 8 and 9 were recombinants, but the sequencing results revealed that they were heterozygous recombinants, which represented most of the recombinants. However, according to the sequencing results, plaques 1, 2, 5, 6 and 10 carried point mutations in the PAM.

**FIG 7:**
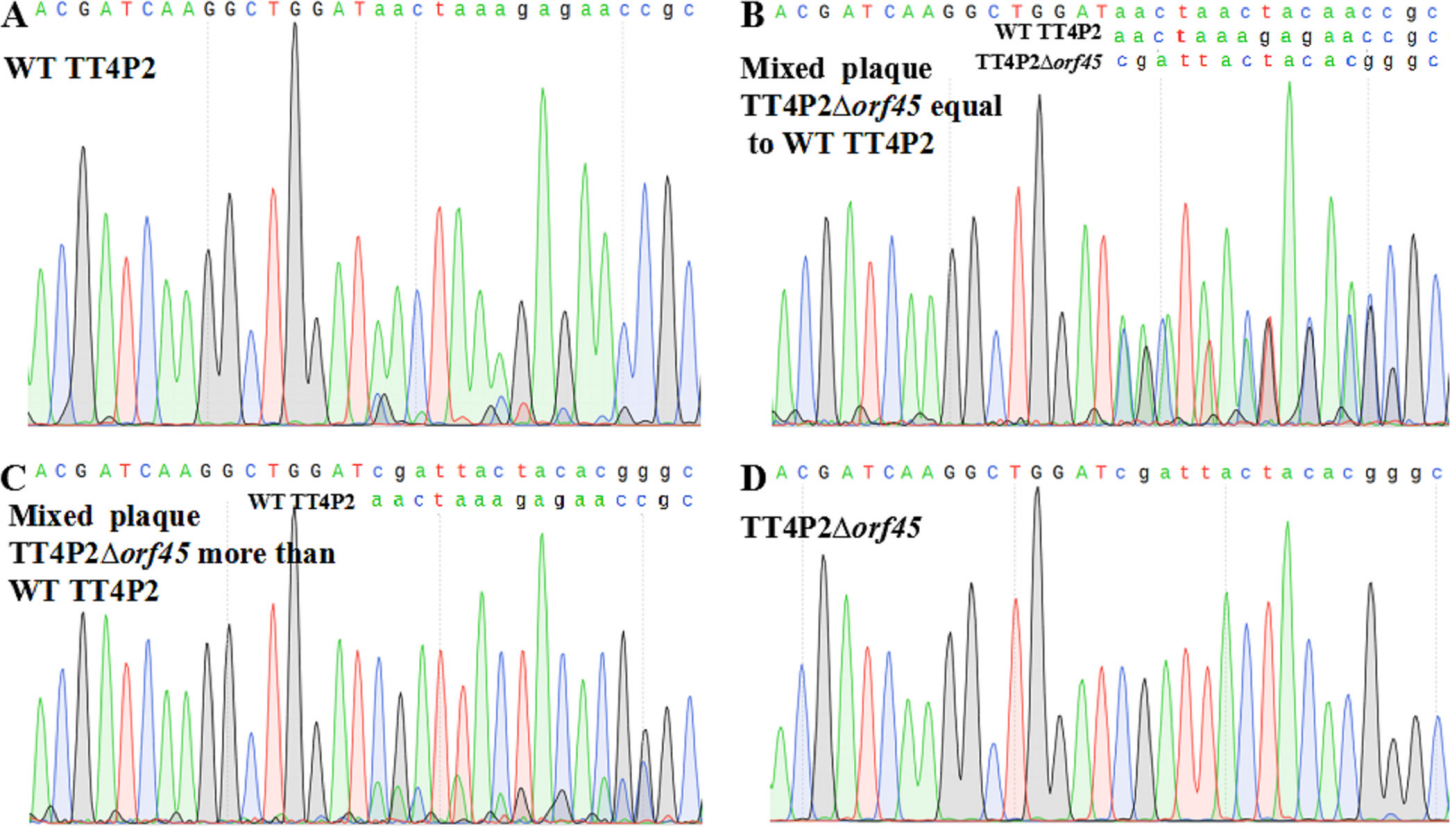
Sequence peaks of 4 plaques of TT4P2*Δorf45*. (A) Sequence peak of the 13th single plaque, which was almost entirely the wild-type phage TT4P2. (B) Peak of the 11th plaque showing that the amount of wild-type phage was approximately equal to that of the recombinant phage TT4P2*Δorf45*. (C) Less wild-type phage than recombinant phage TT4P2*Δorf45* was present according to the peak of the 7th plaque. (D) Most of the 3rd plaques were recombinant phage TT4P2*Δorf45*.

For phage TT4P2-*orf45*, the DPMSR method based on the heterologous CRISPR–Cas9 system was used to perform a gene deletion experiment. In total, 13 plaques were selected and verified by PCR (Fig. 6) and sequencing (Fig. 7). Only 5 plaques were heterozygous recombinants (wild-type and recombinant), and there were no homozygous recombinants. When strain TT4::pT45sp1 was used to further purify the heterozygous recombinant TT4P2*Δorf45*, homozygous recombinant TT4P2*Δorf45* was not always obtained. These results indicated that *orf45* is very likely an essential gene in the life cycle of phage TT4P2. In summary, the two strategies based on the heterologous CRISPR–Cas9 system can be used for phage TT4P2 gene deletion, and the necessity of phage genes can be quickly determined according to the types (homozygous or heterozygous recombinant) of their recombinants.

**(iii) Gene replacement.** From the above experiments, it was determined that *orf6* may be a nonessential gene of phage TT4P2. In addition, genes related to lysis were not identified during genome analysis of phage TT4P2; thus, we tried to apply the SPMAR method based on the heterologous CRISPR–Cas9 system to perform a gene replacement experiment, which used the lysozyme *e* gene of phage EJ(3)9P3-*orf39* to replace the partial fragment (292 bp) of phage TT4P2-*orf6*, and we expected to obtain TT4P2*Δorf6*::e. After two double-layered plate tests, we picked 20 plaques. According to PCR (Fig. S6) and sequencing results, 15 of the 20 plaques were target recombinants TT4P2*Δorf6*::e.

All of the obtained results proved that the two strategies based on the heterologous CRISPR–Cas9 system constructed in *V. natriegens* TT4 could be used to effectively perform phage TT4P2 genome editing experiments (deletion and replacement). Additionally, the necessity of phage genes can be quickly determined according to the types of their recombinants, and the integrity of the foreign plasmid (pTorf-ed and pTorf-sp) in *V. natriegens* TT4 and the activity of SpyCas9:RNA were the keys to screening target recombinants.

## DISCUSSION

Anti-CRISPR genes (50, 52) and CRISPR–Cas systems (19) may exist in the genomes of virulent phages. When the exogenous Streptococcus pyogenes SF370 II-A system (CRISPR–Cas9 system) is used to perform gene editing of phage TT4P2 in *V. natriegens* TT4, it is necessary to determine whether the edited phage harbors anti-Cas9 proteins or an endogenous CRISPR–Cas system, which may cause SpyCas9:RNA inactivity, meaning that it cannot be used as a screening tool (53–55). Compared with three other cases of editing phage genes using the CRISPR–Cas9 system, the effect of SpyCas9:RNA on the random mutation selection of phage target sequences and their PAM in this study was similar to that in the study of Lemay (43), which found almost no point mutations in the target sequence and its PAM among single plaques. However, the subsequent *orf6* deletion experiments in this study showed that the editing efficiency was higher than that in the Lemay study and was similar to those in the studies by Tao (44) and Shen (45), at approximately 100%.

In addition, some studies have shown that the activity of exogenous CRISPR–Cas9 systems is higher, producing phages with lower PFU or EOP. The strain containing the targeted plasmid has a stronger effect on the selection of phage random mutations, which was determined via the double-layered plate experiment combining the strain containing only the targeted plasmid with the phage, resulting in most of the obtained plaques having mutations. Of course, higher immunity may also lead to the failure to form single plaques; therefore, in subsequent experiments, the strain containing the targeted plasmid and its corresponding homologous recombination plasmid had a higher editing efficiency (nearly 100%) for targeted phage genes (40, 42–45).

However, according to research by Lemay (43) and Shen (45) and the results of this study, if the activity of exogenous SpyCas9:RNA is not high and the strain containing the targeted plasmid has a lower effect on the selection of random mutations, then the editing efficiency of the double-plasmid strain for the targeted phage gene depends on the specific situation. For example, the editing efficiency of the nonessential gene *orf6* in this study was nearly 100%, and the mutants were all homozygous. When Shen (45) used low-expression sgRNA-targeted plasmids (proNT1 or proNT2) and their homologous recombination plasmids to perform deletion experiments (approximately 500 bp) on the nonessential genes of phage phiKpS2, the picked plaques were all heterozygous, and wild-type phages accounted for the majority. Lemay (43) performed a deletion experiment for p2-*orf47* (76 bp) in Lactococcus lactis MG1363 containing the targeted plasmid pL2Cas9-47 and the homologous recombination plasmid pKO47, and only 1/3 of plaques were homozygous with the target band, while the other two were heterozygous with two bands. However, phage genome editing that inactivates essential genes generally has difficulty yielding homozygotes (14, 45), and special methods must be used. For example, a complementation assay is used to express the edited gene (deleted) from a plasmid introduced into the host (14), and the use of a suppressor gene (*sup*) to mutate the host bacteria allows full-length translation of the edited gene (nonsense mutation) (44).

In fact, if the edited gene is not essential, the EOP or PFU of the phage obtained by the double-plasmid (targeted plasmid and homologous recombination plasmid) strain will be higher than that of the single-plasmid strain (targeted plasmid). The reason for the EOP increase is that homologous recombination causes double-strand breaks (DSBs) produced in the genome by the CRISPR–Cas system to be respliced in a certain manner, generating target recombinants that can escape the CRISPR–Cas system (39, 40, 42, 44, 45). If the edited gene is necessary, the EOP or PFU obtained from the double-plasmid strain will be lower than that from the single-plasmid (targeted plasmid) strain (45). Moreover, when the activity of the SpyCas9:RNA complex is higher, the plaque obtained from the single-plasmid (targeted plasmid) strain and its mutations generally carries synonymous mutations or missense mutations that have little effect on gene activity (39).

Molecular genetics, synthetic biology, nanobiotechnology (such as phage display technology (56)), pathogenic mechanisms of bacterial diseases, and food safety (47) associated with phages have been broadly explored over the past decades. Phage genome editing technology based on the CRISPR–Cas system, the *de novo* synthesis of phage genomes, and the assembly of phage DNA fragments in yeast (57) may become powerful tools for analyzing and utilizing phage resources, which can also promote the potential application of phage genome editing in synthetic biology. The construction of the *V. natriegens* phage genome editing platform based on the heterologous CRISPR–Cas9 system in this article will provide an example for phage genome editing, especially for *Vibrio* phages genome editing. Moreover, this system lays the foundation for the next steps in deep phage genome transformation, including the simplification of the phage genome, and the collection of dominant phage genes to construct multifunctional phages; additionally, this system is expected to solve the problem of insufficient research on phage gene diversity and even promote the discovery of new molecular biology tools (47).

## MATERIALS AND METHODS

### Bacterial strains, bacteriophages, and culture conditions.

*V. natriegens* TT4 and *V. natriegens* phage TT4P2 were identified and preserved in our laboratory. The experimental strain *V. natriegens* TT4 was removed from the −80°C freezer and first cultured in liquid. Then, the target strain was purified by a dilution coating plate method and multiple streak separations, and finally, the strain was verified by 16S rDNA analysis. The preserved *V. natriegens* phage TT4P2 was cultured in liquid medium together with the strain *V. natriegens* TT4, which had been verified and purified, and then, the phage was purified via a double-layered plate experiment. TT4 and its derivative strains were cultured at 37°C or 30°C with shaking at 200 rpm. Phage TT4P2 and its mutants were cultured at 37°C or 22°C with shaking at 200 rpm. The most common growth media used were Luria–Bertani (LB) broth (supplemented with 1% NaCl) and brain heart infusion (BHI) broth (supplemented with 204 mM NaCl, 4.2 mM KCl, and 23.14 mM MgCl_2_) (48). To prepare solid medium, 2% agar was added to LB. For the double-layer plaque assays, the upper layer was composed of 0.6% agar. Chloramphenicol was added to the medium at a final concentration of 5 μg*/mL* for the selection of TT4 containing the targeted plasmid pTorf-sp. Kanamycin was added to the medium at a final concentration of 130 μg*/mL* (solid) or 350 μg*/mL* (liquid) for the selection of TT4 containing the recombination template plasmid pTorf-ed.

### Preparation of electrocompetent cells and the electrotransformation protocol.

Competent *V. natriegens* TT4 cells were prepared according to Weinstock’s report. The plasmids pTorf-ed and pTorf-sp were electroporated into TT4, which lacks a natural CRISPR–Cas system, at 900 V in a 1 mm cuvette, yielding the recombination template strain and the phage-resistant strain. The cells were recovered by incubation at 37°C for 2 h. Approximately 500 μL of recovered cells was coated onto plates containing the appropriate antibiotic. The plates were incubated overnight at 37°C for colony growth.

### Construction of the targeted plasmid pTorf-sp.

The plasmid pCas9 carrying the Cas9 endonuclease and sgRNA-binding site was purchased from Addgene (plasmid number 42876) to construct all targeted plasmids. The targeted gene sequence of phage TT4P2 was examined to search for a protospacer-adjacent motif (PAM; 5′-NGG-3′), and the upstream sequence (30 bp) of the PAM was the targeted spacer. Synthetic oligonucleotides (Table S4) of the targeted spacers were annealed and ligated into the plasmid pCas9 digested with the *Bsa*I restriction enzyme to form the targeted plasmid pCas9-orf-spacer, named pTorf-sp (Table S3 in the supplemental material). The ratio of insert to vector was 1:2.

### Construction of the recombination template plasmid pTorf-ed.

A recombination template plasmid was established using gene splicing by overlap extension (SOE) PCR to facilitate precise genome editing. The plasmid pCRISPR purchased from Addgene (plasmid number 42875) was used to construct all recombination template plasmids for targeted genes. First, using the genome of phage TT4P2 as a template, the left arm was amplified with the primers torf-p1/p2 (Table S5), and the right arm was amplified with the primers torf-p3/p4 (Table S5). The PCR program was as follows: 94°C for 2 min; 30 cycles of 98°C for 10 s, 55°C for 10 s, and 72°C for 5 s; and 72°C for 5 min. Second, using the second PCR products of the left and right arms as templates, the recombination template was amplified with the primers torf-p1/p4 (Table S5), and the program was as follows: 94°C for 5 min; 35 cycles of 94°C for 30 s, 55°C for 30 s, and 72°C for 1 min; and 72°C for 10 min. The recombination template was digested and ligated into pCRISPR linearized with MluI and XhoI (*orf6*) or *Bln*I and XhoI (*orf45*), resulting in the recombination template plasmid pCRISPR-orf-editing, which was termed pTorf-ed (Fig. S2–S4).

### Stability analysis of the foreign plasmids in *V. natriegens* TT4.

According to the methods of Garneau (49) and Hynes (50), strains containing different exogenous plasmids were cultured in liquid LB medium at 37°C and 200 rpm for 24 h. Then, these strains were transferred in turn and cultivated to the 7th generation, and the bacterial suspension (6 μL per point) of each strain from the 0^th^ to 7th generations was selected and spot seeded onto an LB plate containing the corresponding antibiotics and incubated at room temperature for 22 h. Finally, the experimental results were observed and recorded.

### Gene editing of *V. natriegens* phage TT4P2.

**(i) Double-layered plate experiment.** All plaques were screened by a double-layered plate experiment. First, 0.2 mL of a properly diluted phage suspension and 0.6 mL of a bacterial culture were added to 3.5 mL of warm soft agar (0.6%) supplemented with chloramphenicol or kanamycin, which was then mixed and poured onto solid medium to obtain double-layered plates. The double-layered plates were incubated at room temperature for 12 h to 16 h to form plaques. Well-isolated individual plaques were picked and transferred to 1.5 mL microcentrifuge tubes containing 500 μL of SM solution and 50 μL of chloroform.

**(ii) Immunity of strains to bacteriophage.** Referring to the method of Bondy-Denomy (51), the bacterial suspensions of strains containing the plasmid pTorf-sp or double plasmids were sampled in aliquots of approximately 400 μL to prepare a double-layered plate, and then the diluted solution of phage TT4P2 was spotted onto the double-layered plate with the corresponding antibiotics at 4 μL per spot and incubated at room temperature for 12 h to 16 h. Finally, the experimental results were observed and recorded.

**(iii) Random mutant screening experiment.** Approximately 400 μL of bacterial suspension of strains containing the plasmid pTorf-sp was combined with the appropriately diluted solution of phage TT4P2 to carry out a double-layered plate experiment. After single plaques grew on the double-layered plate, several single plaques were picked, and the primers t6Y-F/R and TT45-F/R1 (Table S5) were used to perform PCR amplification.

**(iv) Gene deletion.** As shown in Fig. 1 (taking DPMSR as an example), strains containing the recombination template plasmid pTorf-ed and the targeted plasmid pTorf-sp were infected with 10-fold serial dilutions of the wild-type phage TT4P2 at a ratio of 200 μL:600 μL (phage:bacterium), and double-layered plate experiments were performed. On the next day, all recombinants from the double-layered plate were screened. Using the primers t6p1/p4, t6Y-F/R and t45Y-F/R (Table S5 in the supplemental material), the accuracy of all recombinants was confirmed by PCR and sequencing.

**(v) Gene replacement.** Gene replacement experiments were performed based on the SPMAR method. The process was as follows: TT4::pT6ed-*e* was infected with 10-fold serial dilutions of the wild-type phage TT4P2, and a double-layered plate experiment was performed. On the following day, a mixed plaque containing the wild-type phage and recombinant phage was screened from the double-layered plate. Second, TT4::pT6sp1 or TT4::pT6sp2 was infected with the corresponding 10-fold serial dilutions of the random mixed plaque, and then another double-layered plate experiment was carried out. On the next day, all recombinants were screened from the double-layered plate. Using the primers ep1/ep4 (Table S5 in the supplemental material), the accuracy of all recombinants was confirmed by PCR and sequencing.
