# Supplementary material for: CRISPR–Cas9 Based Bacteriophage Genome Editing
Source: Microbiol Spectr. 2022 Jul 26;10(4):e00820-22. doi: 10.1128/spectrum.00820-22 (PMC9430736; doi:10.1128/spectrum.00820-22)
Supplement: Supplemental file 1 — Supplemental material. Download spectrum.00820-22-s0001.pdf, PDF file, 0.5 MB [file spectrum.00820-22-s0001.pdf]

SUPPLEMENTARY DATA

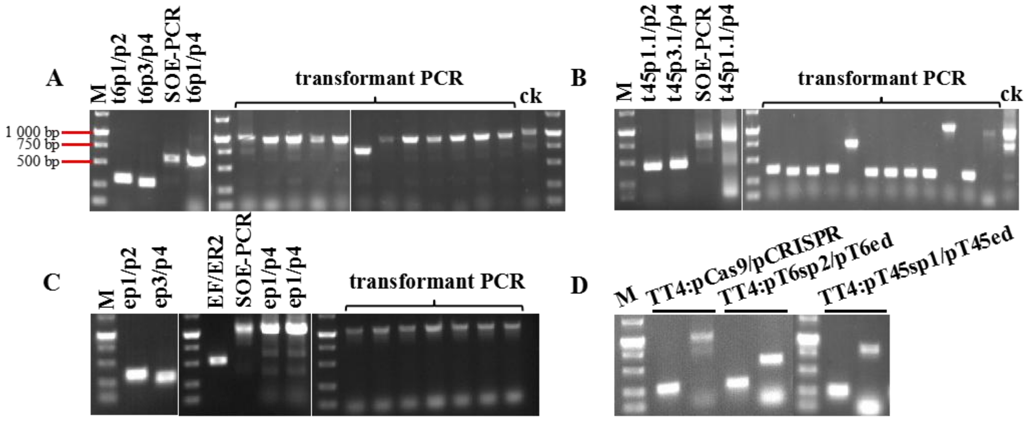

FIG S1 Construction of strains with recombination template plasmid, or recombination template and targeted plasmids. M is DL5000 DNA Marker. The vertical text in the figure illustrats the primers used in PCR, except for SOE-PCR, which was the result of the second round of SOE-PCR (gene splicing by overlap extension- PCR). (A and B) The construction of TT4::pT6ed and TT4::pT45ed, respectively. The primers used in the PCR of transformants were pCRISPR-F/R. Ck is the result of TT4::pCRISPR amplified with primer pCRISPR-F/R. (C) The construction of TT4::pT6ed-e. Gene *e* was amplified with primers EF/ER2. The primers used for PCR of transformants were ep1/p4. (D) The verification of the strains containing double plasmids. Plasmid pCas9 was verified with primers DR-F/R. pCRISPR, pT6ed and pT45ed were verified with primers pCRISPR-F/R, t6p1/p4 and t45p1.1/p4, respectively.

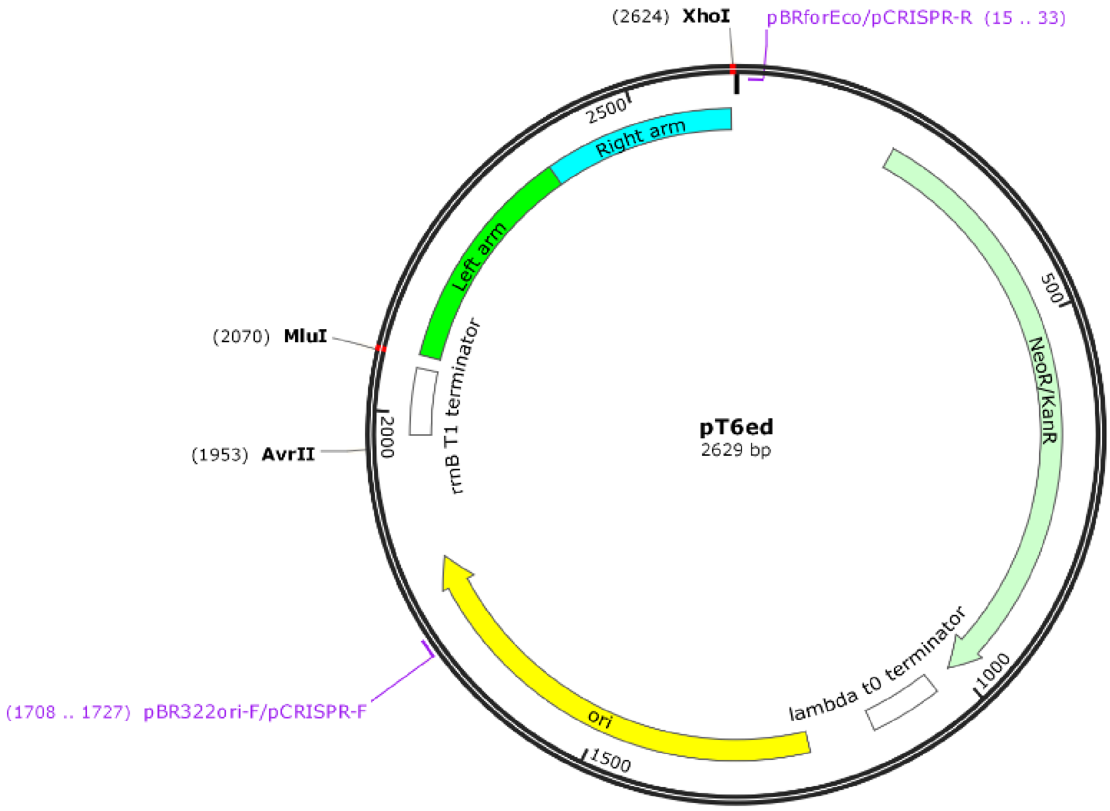

FIG S2 Map of plasmid pT6ed. The left and the right arm were the gene sequence of phage TT4P2, which can be linked to plasmid pCRISPR after enzyme digestion (*Mlu*I and *Xho*I marked with red) to construct homologous recombination plasmid pT6ed. The plasmid pT6ed can promote the homologous recombination of phage TT4P2, when performed the deletion of phage TT4P2-*orf6*.

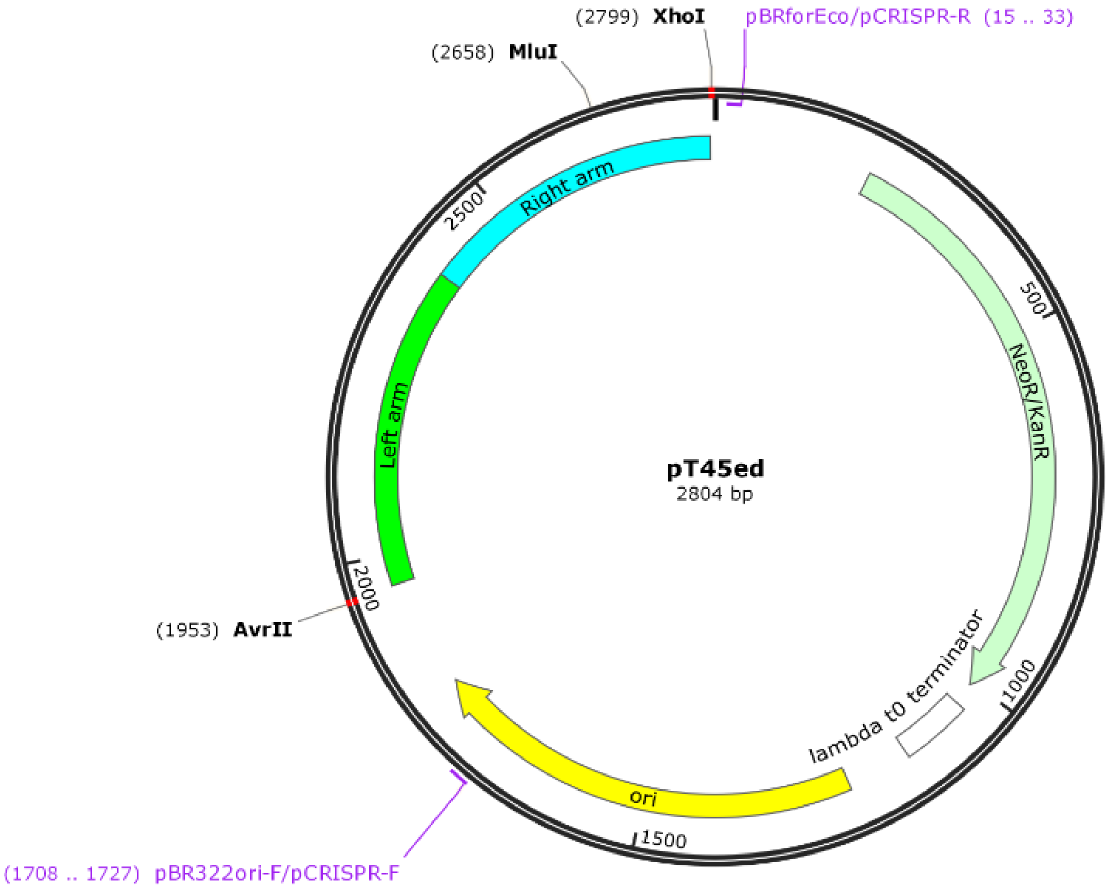

FIG S3 Map of plasmid pT45ed. The left and the right arm were the gene sequence of phage TT4P2, which can be linked to plasmid pCRISPR after enzyme digestion (*Avr*II and *Xho*I marked with red) to construct homologous recombination plasmid pT45ed. The plasmid pT45ed can promote the homologous recombination of phage TT4P2, when performed the deletion of phage TT4P2-*orf45*.

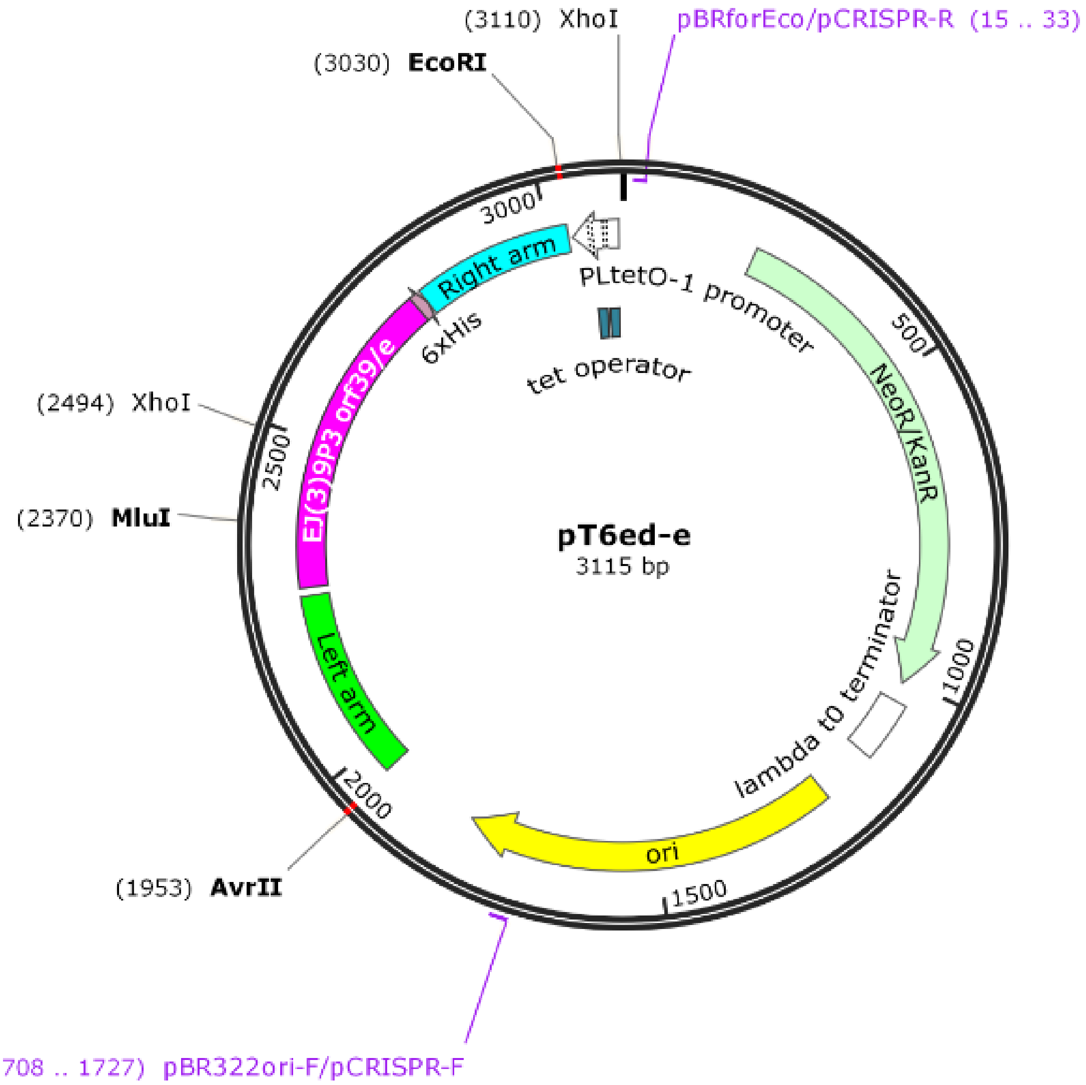

FIG S4 Map of plasmid pT6ed-e. The left and the right arm were the gene sequence of phage TT4P2, which can be linked to plasmid pCRISPR after enzyme digestion (*Avr*II and *Eco*RI marked with red) to construct plasmid pT6ed-e. EJ(3)9P3-*orf39* is lysozyme *e*. The plasmid pT6ed-e was used for the gene replacement between TT4P2-*orf6* and EJ(3)9P3-*orf39*.

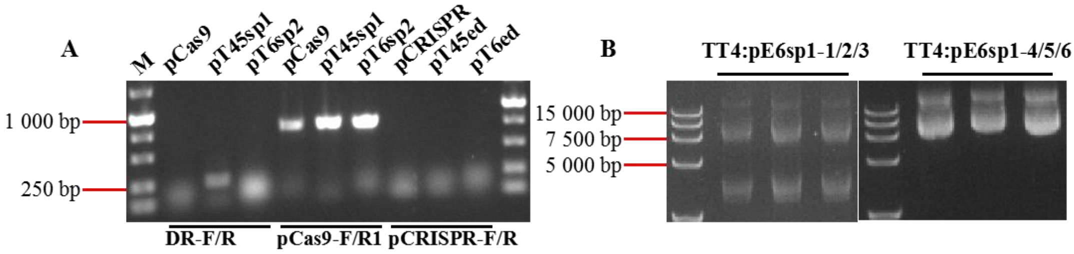

FIG S5 PCR of bacterial suspensions and extraction of pE6sp1 for plasmid stability Assay. M is DL5000 DNA Marker. (A) PCR results of plasmids were used to verify their stability. The labels below the picture were primers. (B) The extraction of pE6sp1. A sequence in pCas9 was replaced with a spacer1 sequence targeting EH(3)23P1-*orf6* to form plasmid pE6sp1. Plasmids were extracted from TT4::pE6sp1. 1/2/3 were extracted from TT4::pE6sp1, which was taken out at -20 ℃ and directly subcultured. 4/5/6 were extracted from TT4::pE6sp1, which was taken out at -20 ℃ and streaking on the plate to picked single colony.

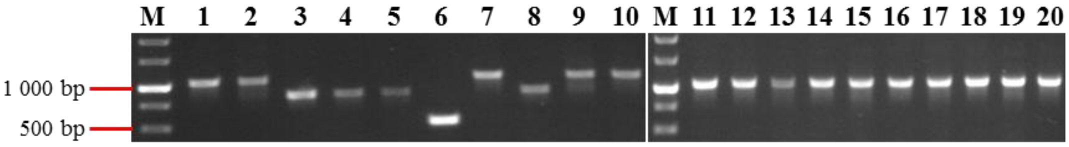

FIG S6 Replacement of phage TT4P2-*orf6* with lysozyme gene *e* of phage EJ(3)9P3 (*orf39*). M is DL5000 DNA Marker. 1-20 were plaques. The first 10 plaques were picked from TT4::pT6sp2, and the last 10 plaques were picked from TT4::pT6sp1. The plaques of 3, 4, 5 and 8 were wild-type. The plaque of 6 was a nontarget recombinant.

TABLE 1 Strains used in this study

Strain	Source or Reference
Vibrio natriegens TT4	Preserved in this laboratory
Enterobacter spp. EJ(3)9	Preserved in this laboratory
Escherichia coli DH5α::pCas9	Addgene
E. coli DH5α::pCRISPR	Addgene
V. natriegens TT4::pCas9	TT4 contains plasmid pCas9
V. natriegens TT4::pCRISPR	TT4 contains plasmid pCRISPR
V. natriegens TT4::pCas9/pCRISPR	TT4 contains plasmid pCas9 and pCRISPR
V. natriegens TT4::pT6sp1	TT4 contains plasmid pT6sp1
V. natriegens TT4::pT6sp2	TT4 contains plasmid pT6sp2
V. natriegens TT4::pT6ed	TT4 contains plasmid pT6ed
V. natriegens TT4::pT6ed-*e*	TT4 contains plasmid pT6ed-*e*
V. natriegens TT4::pT45sp1	TT4 contains plasmid pT45sp1
V. natriegens TT4::pT45sp2	TT4 contains plasmid pT45sp2
V. natriegens TT4::pT45ed	TT4 contains plasmid pT45ed
V. natriegens TT4::pT6sp2/pT6ed	TT4 contains plasmid pT6sp2 and pT6ed
V. natriegens TT4::pT45sp1/pT45ed	TT4 contains plasmid pT45sp1 and pT45ed
V. natriegens TT4::pE6sp1	TT4 contains plasmid pE6sp1

Note: In order to select plasmid pCas9 from *E. coli* DH5α::pCas9, chloramphenicol was added to medium at a final concentration of 25μg/ml. The selection of plasmid pCRISPR from *E.coli* DH5α::pCRISPR was added to medium at a kanamycin final concentration of 50μg/ml. *V. natriegens* phage TT4P2-*orf6* is indicated by T6; TT4P2-*orf6*-spacer is indicated by T6sp, which was connected to plasmid pCas9 to form plasmid pT6sp; TT4P2-*orf6*-editing is indicated by T6ed, which was connected to plasmid pCRISPR to form plasmid pT6ed. The same is as TT4P2-*orf45*.

TABLE 2 Bacteriophages used in this study

Bacteriophage	Source or Reference
Vibrio natriegens siphophage TT4P2	Preserved in this laboratory
Enterobacter spp. myophage EJ(3)9P3	Preserved in this laboratory
TT4P2Δ*orf6*	TT4P2 *orf6* deleted 292 bp, constructed in this study
TT4P2Δ*orf5*Δ*orf6*Δ*orf7*	TT4P2 deleted 820 bp, including the entire *orf6*, 121 bp at the back of *orf5*, and 182 bp at the front of *orf7*, constructed in this study
TT4P2Δ*orf6*Δ*orf7*	TT4P2 deleted 347 bp, occurring between two short repeats in the same direction. One of the two repetitive sequences is in *orf6*, and the latter is in *orf7*, constructed in this study
TT4P2Δ*orf6*::*e*	The 292 bp of phage TT4P2-*orf6* was replaced with *orf39*(*e*) of phage EJ(3)9P3, constructed in this study
TT4P2Δ*orf*45	TT4P2 *orf45* deleted 162 bp, constructed in this study

TABLE 3 Plasmids used in this study

Plasmid	Description	Source or Reference
pCas9	Low copy, Cam^R, 9326 bp	Addgene
pCRISPR	High copy, Kan^R, 2707 bp	Addgene
pT6sp1	The original sequence in pCas9 was replaced with a spacer (sp1) targeting TT4P2 *orf6*	This study
pT6sp2	The original sequence in pCas9 was replaced with a spacer (sp2) targeting TT4P2 *orf6*	This study
pT6ed	Transformed by pCRISPR to delete 292 bp of TT4P2 *orf6*	This study
pT6ed-*e*	Transformed by pCRISPR to replace 292 bp of TT4P2 *orf6* with EJ(3)9P3-*orf39*	This study
pT45sp1	The original sequence in pCas9 was replaced with a spacer (sp1) targeting TT4P2 *orf45*	This study
pT45sp2	The original sequence in pCas9 was replaced with a spacer (sp2) targeting TT4P2 *orf45*	This study
pT45ed	Transformed by pCRISPR to delete 162 bp of TT4P2 *orf45*	This study

Note: *V. natriegens* phage TT4P2-*orf45* is indicated by T45; TT4P2-*orf45*-spacer is indicated by T45sp, which was connected to plasmid pCas9 to form plasmid pT45sp; TT4P2-*orf45*-editing is indicated by T45ed, which was connected to plasmid pCRISPR to form plasmid pT45ed.

TABLE 4 Oligos used in this

Spacer oligo	Sequence (5'-3')	Description
t6oligo-sp1 I	AAACTAAACGAAAAGCCCGCTATTGAATCCGTTAG	The spacer (sp1) of TT4P2 *orf6*; Targeting TT4P2 *orf6*
t6oligo-sp1 II	AAAACTAACGGATTCAATAGCGGGCTTTTCGTTTA	
t6oligo-sp2 I	AAACCACGTTTTCGCAGGTTGTAAGATCAACTTAG	The spacer (sp2) of TT4P2 *orf6*; Targeting TT4P2 *orf6*
t6oligo-sp2 II	AAAACTAAGTTGATCTTACAACCTGCGAAAACGTG	
t45oligo-sp1 I	AAACCGACAAGCTACTAGACCTCGCGAGCCGTATG	The spacer (sp1) of TT4P2 *orf45*; Targeting TT4P2 *orf45*
t45oligo-sp1 II	AAAACATACGGCTCGCGAGGTCTAGTAGCTTGTCG	
t45oligo-sp2 I	AAACCCCATTCGTCCATCGTAGAATACCCCTAAAG	The spacer (sp2) of TT4P2 *orf45*; Targeting TT4P2 *orf45*
t45oligo-sp2 II	AAAACTTTAGGGGTATTCTACGATGGACGAATGGG	

Note: The underscore at oligos was the sequence of spacers.

TABLE 5 Primers used in this

Primers title	Sequence (5'-3')	Description
e1/e_pET_ex_his_1	ATAGGAGGTCCCCATGGACATT	Verified EJ(3)9P3
e2/e_pET_ex_his_2	AAATGTCGACTAGGTTTTCATATGC	
pBR322ori-F/pCas9-R1	GGTGATGTCGGCGATATAGG	Verified pCas9
CAT-R/pCas9-F	GCAACTGACTGAAATGCCTC	
DR-F	GCTGAGACAAATAGTGCG	Verified spacer
DR-R	GTATCCGACTGCTGGTATT	
pBR322ori-F/pCRISPR-F	GGGAAACGCCTGGTATCTTT	Verified pCRISPR
pBRforEco/pCRISPR-R	AATAGGCGTATCACGAGGC	
t6p1	TACGACGCGTCATGAGACGCTAGGAGAAC	Delete TT4P2 *orf6*
t6p2	ATGAGTGATCGAAGTGGG	
t6p3	CGCCCACTTCGATCACTCATGCTTATCGCGTT TACTGG	
t6p4	TCTACTCGAGTCCTTTAGGTTGGCTTCG	
t6Y-F	TCCCTGAACCTGACACCAG	Verified TT4P2Δ*orf6*
t6Y-R	TTAGGCTCGCACTGTCCCA	
ep1	TACGCCTAGGCATGAGACGCTAGGAGAAC	Replaced TT4P2*orf6*
ep2	ATGCCAAAAATGTCCATGGGGACCTCCTATA TGAGTGATCGAAGTGGG	
ep3	TATGAAAACCTACATCATCATCATCATCATG CTTATCGCGTTTACTGG	
ep4	TCTAGAATTCTCCTTTAGGTTGGCTTCG	
EF/e1	ATAGGAGGTCCCCATGGACATT	
ER2	ATGATGATGATGATGATGTAGGTTTTCATAT GCTTTCC	
t45p1.1	TACGCCTAGGTGAACGCGATTAACTACGG	Delete TT4P2 *orf45*
t45p2	ATCCAGCCTTGATCGTTG	
t45p3.1	CCTGACAGCAACCAACGATCAAGGCTGGAT CGATTACTACACGGGCACT	
t45p4	TCTACTCGAGCAACGTATGACGCTCCAA	
t45Y-F	CGCCAGAAATTAACACGC	Verified TT4P2Δ*orf45*
t45Y-R	AAGCCCAAACACCCTCAA	
TT45-F	GATGAACTACGCAGGGAT	
TT45-R1	GGTGTGATTGCCATATAAAG	

Note: The underline at 6 bases was restriction endonuclease cleavage site. The underline at p2/p3 was the complementary sequence in overlap extension PCR. The wavy line at ER2 was tag of 6 ×His.
